# Behavioral decision-making of government, agricultural product producers, and consumers on agricultural product quality and safety regulation in a digital environment

**DOI:** 10.3389/fpubh.2024.1373747

**Published:** 2024-04-02

**Authors:** Hong Huo, Xiangyu Liu

**Affiliations:** Management School, Harbin University of Commerce, Harbin, China

**Keywords:** agricultural product quality and safety, digital traceability system, quality and safety supervision, game evolution, public health

## Abstract

The quality and safety of agricultural products are related to people’s lives and health, economic development, and social stability, and have always been a hot issue of concern to the government and society. The rapid development of digital traceability technology in the digital environment has brought new opportunities for the supervision of agricultural product quality and safety, but the frequent occurrence of agricultural product safety incidents in recent years has exposed many problems such as the lack of governmental supervision, unstandardized production process of enterprises, and weak consumer awareness. To improve the cooperation efficiency of stakeholders and ensure the quality and safety of agricultural products, this paper proposes a dynamic model based on evolutionary game theory. The model incorporates the government, agricultural product producers, and farmers, and evaluates the stability and effectiveness of the system under different circumstances. The results of the study show that there are multiple evolutionary stabilization strategies in the tripartite evolutionary game model of agricultural product quality and safety supervision, and there are corresponding evolutionary stabilization conditions. There are several factors affecting the stability of the system, the most important of which are government regulation, severe penalties for agricultural product producers, and incentives. When these factors reach a certain threshold, the stakeholder cooperation mechanism can establish an evolutionarily stable strategy. This study contributes to the understanding of the operational mechanism of stakeholder cooperation in agricultural product quality and safety regulation in the digital environment and provides decision support and policy recommendations for stakeholders to promote the sustainable development and optimization of agricultural product quality and safety regulation.

## 1 Introduction

The quality and safety of agricultural products are the foundation of food quality and safety, which is related to people’s health and life safety, and has a bearing on farmers’ income increase and the high-quality development of agriculture and rural areas (1, 2). Ensuring the quality and safety of agricultural products has become an important challenge as the demand for agricultural products increases year after year with the growing population and accelerated urbanization (3, 4). With the continuous progress of science and technology and the popularization of information technology, digital technology has brought new opportunities for supervising agricultural quality and safety (5), such as real-time monitoring and collection of agricultural product quality data, and agricultural product quality traceability based on blockchain. However, there have been frequent incidents of quality and safety of all kinds of agricultural products (6, 7), such as the “stale grain” incident, the “Sudan red incident” (8), the “malachite green incident,” the “artificial honey” incident in Wuhan, Hubei, and other places, and so on. These events have exposed that digital traceability technology has not been effectively used in the supervision of agricultural product quality and safety, and how to apply digital technology to the supervision of agricultural product quality and safety has become an urgent problem to be solved (9).

The process of monitoring the quality and safety of agricultural products involves the government, agricultural production enterprises, consumers, and other multi-interested parties (10, 11). Scholars at china and abroad have conducted some studies on the roles played by various subjects in the supervision of agricultural product quality and safety. The government plays a leading role in the process of regulating the quality and safety of agricultural products and is involved in the complete industrial chain of agricultural products from field to table (12, 13). Teng et al. (14) argue that effective government regulation can promote farmers’ green production behavior. According to Bhatt, the regulation of agricultural product trading has gradually tended to be government-led (15), with coordinated social supervision covering the media, consumers, the general public, and even farmers and enterprises. Agricultural production enterprises, as important providers of agricultural products, also play an important role in the management of agricultural product quality and safety (16, 17). Enterprises, as a fit between business and information flows, have different advantages in food safety and quality control practices (18). Lezoche et al. (19) believe that the core enterprises of the agricultural supply chain have an important position in the agricultural supply chain, can influence other members of the supply chain to maintain a dynamic cooperation mechanism, and play an important role in quality and safety management (20). Consumers, as direct stakeholders (21), have become important participants in the supervision of agricultural product quality and safety (22). Introducing consumer participation in governance and giving full play to the power of consumer groups can effectively alleviate the problem of insufficient regulatory resources and help eliminate regulatory blind spots, which is an inevitable choice for social co-governance of food safety (23).

Traceability is vital in food quality and safety management (24). With the deep integration of digital technologies such as the Internet of Things, big data, cloud computing, blockchain, and other digital technologies with intelligent agriculture (2, 25), the digital agricultural product traceability system provides a new way of thinking for the supervision of agricultural product quality and safety (26). Consumers are also more inclined to buy traceable produce (21). Establishing the digital agricultural product traceability system requires the joint participation of the government, agricultural product producers, processors, inspection and certification organizations, consumers and other main bodies, which is indispensable.

An evolutionary game model is a mathematical model that uses the principles of evolution and the framework of game theory to study the interactions between individuals in a biological population (27). The model aims to explore how the frequency of different types of individuals in a population changes over time and how this change is affected by interactions between individuals and environmental influences (28, 29). Evolutionary game models are often used by scholars to discuss the interrelationships between the three parties in an interaction. For example, in environmental monitoring, Encarnação et al. (30) develop a new framework based on evolutionary game theory, envisioning that the state, business and civil sectors are faced with the dilemma of deciding between maintaining the status quo or shifting to a new paradigm, and the results show that public intervention is essential for shifting to a new paradigm, and that synergies between the private and civil sector are an important step in supporting the paradigm shift. In Healthcare Investing, Alalawi et al. (31) provide a theoretical and simulation analysis of healthcare business models involving Public Healthcare Providers, Private Healthcare Providers and Patients, contributing to the modeling of the healthcare economy by analyzing the dynamics of agents and the emergence of collaborative behaviors in the three populations. Bova et al. (32) use evolutionary game models to explore the role governments can play in building regulatory markets for AI systems to prevent reckless behavior. So it can be concluded from previous studies that the evolutionary game model can help us analyze the conflict of interest and cooperation between different stakeholders (33). In our research, the field of agricultural product quality and safety supervision involves multiple stakeholders (34), and the evolutionary game model helps to reveal the complex game relationship among them. Simulating the strategy selection and decision-making process of each participant helps to optimize the regulatory system and improve the quality and safety of agricultural products (35). In the regulation of agricultural product quality and safety, information asymmetry often leads to increased difficulty in regulation, and the dynamic process of information transfer and gaming can be better understood using evolutionary game models (36). The evolutionary game model can predict the behavioral evolution paths of different players in different contexts (37), which helps to formulate more effective regulatory strategies and countermeasures and improve regulatory effectiveness quality and safety levels.

Therefore, scholars have widely used game theory in the study of quality and safety control of agricultural products (38, 39). Chen et.al (40) analyzed input capacity constraints’ impact on food quality and quality regulation through game theory. Based on the evolutionary game theory, a game model between the government, farmers, and consumers was established, and the results showed that the government subsidy strength, to farmers, consumer trust coefficient, and willingness to pay the premium for carbon-labeled agricultural products were positively correlated with the adoption of low-carbon production behavior by farmers. Chen et al. (41) introduced the social preference theory to construct an evolutionary game model among multiple subjects and studied how to guide the behavioral decisions of multiple subjects to be standardized and rationalized (42). Teng et al. (14) studied the evolutionary decision-making behavior of government, farmers, and consumers based on the perspective of agricultural product quality and safety. Ma et al. (43) constructed a three-party evolutionary game model of consumers, government, and farmers in the context of COVID-19 prevention and control normalization. The results showed that the cost of government regulation, the evaluation of the government by consumers and pig farmers, the government’s subsidies to pig farmers and consumers, and the proportion of stakeholder behaviors affect the formation of the three-party relationship.

Some scholars have used evolutionary game theory to model incentives and agreement compliance. For example, Li et al. (44) established an evolutionary game model of the governance mechanism of the recycling industry and analyzed the impact of government punishment on the behavioral strategies of recycling firms. Sasaki et al. (45) combine key aspects of characterizing different punishment mechanisms in an evolutionary game-theoretic perspective and introduce a strategy of simultaneous commitment to cooperation and peer punishment to investigate a new mechanism for maintaining social order. Starting from a game-theoretic model that captures hegemonic competition in the field using artificial intelligence techniques, Han et al. (46) show how sanctions, when applied unconditionally to potentially unsafe behaviors, may produce socially undesired outcomes. With the help of evolutionary game theory, Ogbo et al. (47) analyze how ex-ante commitment can enhance the coordination of parties in two-by-two and multi-party interactions when the outcome presents an asymmetric payoff structure. Barrett uses a simple game model to illustrate whether and how treaties and related institutions can change incentives to align national self-interests with collective interests (48). Han, on the other hand, shows that evolutionary game theory provides a suitable tool for studying the evolution of cooperative behaviors in social dilemmas as they are governed by institutional incentives and prior commitments (49). Therefore, this paper also adopts evolutionary game theory to discuss the dynamic reward and dynamic punishment mechanism under the participation of multiple actors in the supervision of agricultural product quality and safety.

Some scholars have also taken digitization into account. Wan (50) applies big data technology to the governance of agricultural product quality and safety and utilizes extensive data methods to study the critical control points in the traceability process of agricultural products. Considering the altruistic reciprocity of supermarkets and the fairness concern of processors, respectively, Qin et al. (51) placed the Stackelberg game model under the Corporate social responsibility of processors and investigated the effects of Corporate social responsibility, altruistic reciprocity, and fairness concern on the quality improvement of agricultural supply chains.

In summary, most of the existing studies are on traditional agricultural product quality and safety supervision, and some scholars have studied agricultural product quality and safety traceability and governance from the perspective of big data technology. Few scholars have applied evolutionary game models to agricultural quality and safety regulation in digital environments. Therefore, this paper adopts the evolutionary game method to study the behavioral strategies of the government, agricultural product producers, and consumers in the process of agricultural product quality and safety supervision under the digital environment, analyze the mutual influence of strategic choices between different subjects, and provide theoretical references for the construction of the digital agricultural product traceability system, to further improve the quality and safety of agricultural products, safeguard the rights and interests of consumers, and continuously maintain the stability of the social order.

## 2 Model assumptions and construction

### 2.1 Model assumptions

In this paper, we choose three subjects: government, agricultural producers, and consumers, and put forward the following hypotheses:

1. The government is Participant 1, the agricultural producer is Participant 2, and the consumer is Participant 3. All three participants are assumed to be finite-rational and aim to maximize profit.
2. The government’s strategic choice is to “Regulate” or “Not regulate.” Assume that the probability that the government chooses to “regulate” is x (0<x<1), and the probability that it chooses “Not regulate” is 1-x. When the government chooses to “Regulate,” it will incur the cost of regulation, but it will also enhance the government’s image and trust, and gain certain benefits; when the government chooses “Not regulate,” it will not incur the cost of regulation, but it will lower the government’s image and trust, and incur the certain loss of trust.
3. The strategic choice of the agricultural producer is “Build” or “Not build”. Assume that the probability that an agricultural enterprise chooses to “build” is y (0<y<1), and the probability that it chooses to “not build” is 1-y. When an agricultural producer chooses to “building,” i.e., construct a digital traceability system for agricultural products, it will incur the cost of construction, but the government will provide incentives, and at the same time improve the reputation and trust of the enterprise; When an agricultural product manufacturer chooses “not build”, i.e., chooses not to build a digital traceability system for agricultural products, it will save the technical, human and financial costs of building a traceability system, but it will be penalized by the government accordingly, and at the same time, it will also reduce the reputation and trust of the enterprise.
4. The consumer’s strategic choice is “satisfied” or “dissatisfied”. Assume that the probability that the consumer chooses “satisfied” is z (0<z<1), then the probability that the consumer chooses “dissatisfied” is 1-z. When consumers are “satisfied” with the products produced by agricultural product manufacturers and the operation of the digital agricultural product traceability system, they will receive corresponding economic, health and environmental benefits. When consumers are “dissatisfied” with the products produced by agricultural product manufacturers and the operation of the digital agricultural products traceability system, they will incur corresponding rights defense costs. In contrast, agricultural product manufacturers will incur corresponding losses.

**Figure 1:**
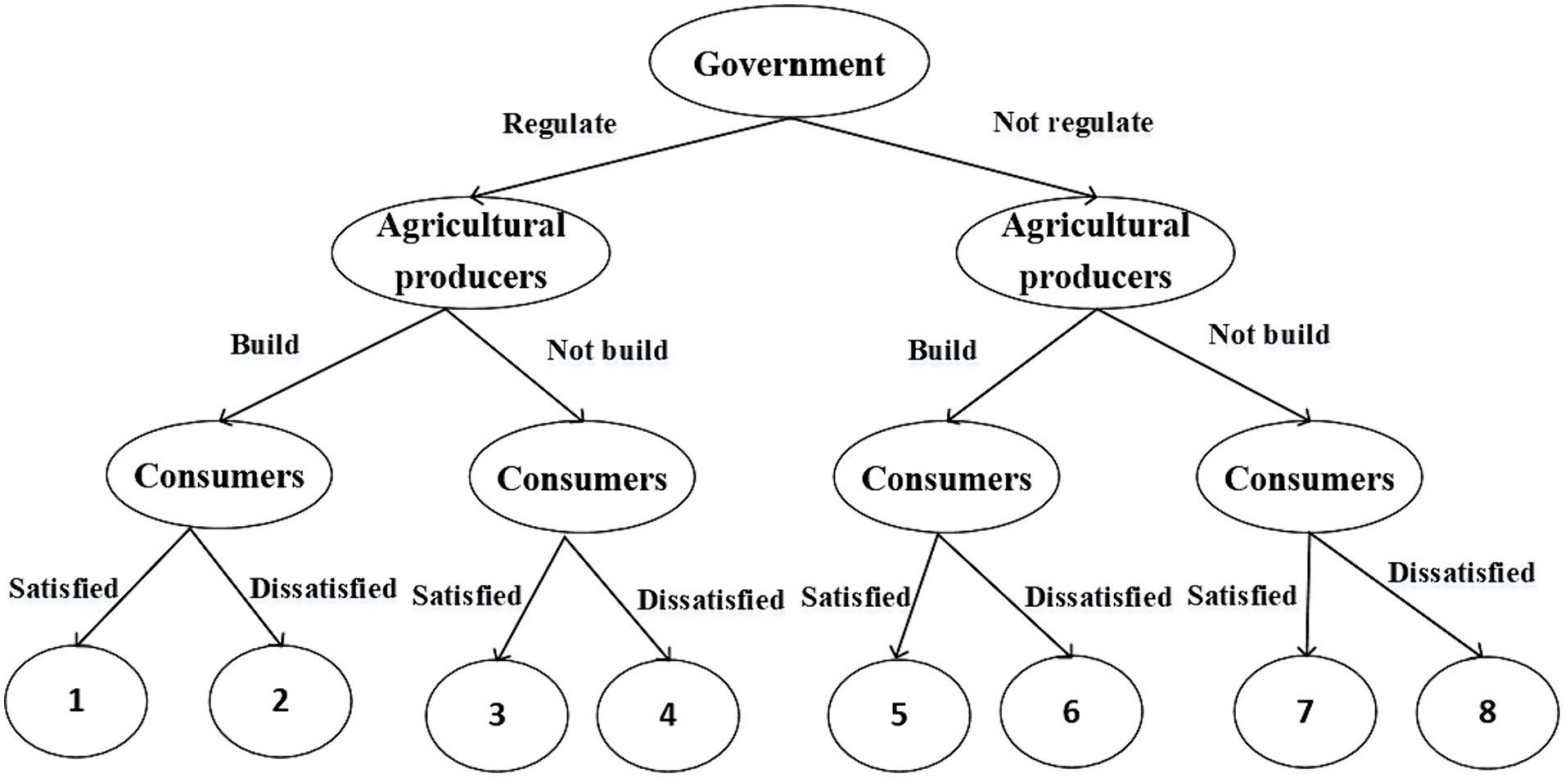
Gaming tree.

Based on the above, the game process between the government, agricultural producers, and consumers is visualized by building a game tree, as shown in Figure 1.

### 2.2 Model construction

#### 2.2.1 Parameter setting

#### 2.2.2 Payment matrix

According to the parameter settings in Table 1, the payment matrix of the three parties of the game is constructed, as shown in Table 2.

**Table 1:** Related parameter settings.

Parameter name	Parameter description
$C_{1}$	Costs incurred by Governments choosing to “Regulate”.
$R_{1}$	Gains from the Government’s choice to “Regulate”.
$L_{1}$	Loss of trust resulting from the Government’s choice to “Not regulate”.
$C_{2}$	Costs incurred by agro-producing firms choosing to “Build”.
A	Government incentives for agricultural producers to choose “Build”.
P	Penalties for agricultural producers choosing not to construct.
$R_{2}$	Benefits to consumers when they are “Satisfied” with the produce they purchase.
$C_{3}$	Costs incurred by consumers in purchasing agricultural products.
$C_{4}$	Costs of advocacy when consumers are “Dissatisfied”.
L	Losses incurred by agricultural producers when consumers opt for “Dissatisfied”.

**Table 2:** Payment matrix for the three parties of the game.

Strategic choice			Government	
			Regulate x	Not regulate 1-x
Agricultural producers	Build y	Satisfied z	$R_{1}-C_{1}-A$, $A-C_{2}$, $R_{2}$	$-L_{1}$, $-C_{2}$, $R_{2}$
		Dissatisfied 1−z	$R_{1}-C_{1}$, $A-C_{2}$, $-C_{4}$	$-L_{1}$, $-C_{2}$, $-T_{1}-C_{4}$
	Not build 1−y	Satisfied z	$R_{1}-C_{1}+P$, −P, $R_{2}$	$-L_{1}$, 0, $R_{2}$
		Dissatisfied 1−z	$R_{1}-C_{1}+P$, $-P-L-C_{4}$, $-C_{3}-C_{4}$	$-L_{1}$, 0, $-L-L_{1}-C_{3}$

## 3 Model analysis

An evolutionarily stable strategy is a strategy in a population that maintains a high degree of adaptability in the face of different strategies and cannot be replaced by other strategies (52). This strategy maintains its dominant position in the group through stability and non-invasiveness, and remains relatively stable during the evolutionary process. We search for more stable strategies by building game models and analyzing the evolution of strategies.

### 3.1 Government’s evolutionarily stable strategy

The expected returns to government regulation and non-regulation$E_{11}$, $E_{12}$, and the average expected return ${\bar{E}}_{1}$ are:


$$\begin{array}{l} \begin{array}{l} E_{11}=yz\left(R_{1}-C_{1}-A\right)+y\left(1-z\right)\left(R_{1}-C_{1}\right)+z\left(1-y\right)\left(R_{1}-C_{1}+P\right)\\ \;+\left(1-y\right)\left(1-z\right)\left(R_{1}-C_{1}+P\right) \end{array}\\ \;=R_{1}-C_{1}-yzA+\left(1-y\right)P \end{array}$$

$$\begin{array}{l} E_{12}=yz\left(-L_{1}\right)+y\left(1-z\right)\left(-L_{1}\right)+z\left(1-y\right)\left(-L_{1}\right)\\ \;+\left(1-y\right)\left(1-z\right)\left(-L_{1}\right)=-L_{1} \end{array}$$

$${\bar{E}}_{1}=xE_{11}+\left(1-x\right)E_{12}=x\left[R_{1}-C_{1}-yzA+\left(1-y\right)P\right]+\left(1-x\right)\left(-L_{1}\right)$$

The replication dynamic equation for government strategy choice is:


$$\begin{array}{l} F\left(x\right)=dx/dt=x\left(E_{11}-{\bar{E}}_{1}\right)=x\left(x-1\right)\\ \;\left[C_{1}-R_{1}+yzA-\left(1-y\right)P-L_{1}\right] \end{array}$$

When $y=\frac{P+L_{1}-C_{1}+R_{1}}{ZA+P}$, $\frac{dF\left(x\right)}{dx}\text{≡}0$, It is in a steady state regardless of the value of x. If $y\neq \frac{P+L_{1}-C_{1}+R_{1}}{ZA+P}$, it is in a steady state at x=0 and x=1.

Perform a derivation of Fx:dFxdx=2x−1C1−R1+yzA−1−yP−L1.

When $y>\frac{P+L_{1}-C_{1}+R_{1}}{ZA+P}$, $\frac{dF\left(x\right)}{dt}|{\;}_{x=1}>0$, $\frac{dF\left(x\right)}{dt}|{\;}_{x=0}<0$, $x^{*}=0$ is an evolutionarily stable strategy. When $y<\frac{P+L_{1}-C_{1}+R_{1}}{ZA+P}$, $\frac{dF\left(x\right)}{dt}|{\;}_{x=1}<0$, $\frac{dF\left(x\right)}{dt}|{\;}_{x=0}>0$, $x^{*}=1$is an evolutionarily stable strategy.

### 3.2 Evolutionary stabilization strategies for agricultural producers

The expected returns of agricultural producers$E_{21}$, $E_{22}$, and the average expected returns ${\bar{E}}_{2}$ are:


$$\begin{array}{l} E_{21}=xz\left(A-C_{2}\right)+z\left(1-x\right)\left(-C_{2}\right)+x\left(1-z\right)\left(A-C_{2}\right)\\ \;+\left(1-z\right)\left(1-x\right)\left(-C_{2}\right)=xA-C_{2} \end{array}$$

$$\begin{array}{l} E_{22}=xz\left(-P\right)+x\left(1-z\right)\left(-P-L-C_{4}\right)\\ \;=x\left(-P\right)+x\left(1-z\right)\left(-L-C_{4}\right) \end{array}$$

$$\begin{array}{l} {\bar{E}}_{2}=yE_{21}+\left(1-y\right)E_{22}=y\left(xA-C_{2}\right)+\\ \;\left(1-y\right)\left[x\left(-P\right)+x\left(1-z\right)\left(-L-C_{4}\right)\right] \end{array}$$

The replication dynamic equation for the strategy choice of agricultural producers is:


$$\begin{array}{l} F\left(y\right)=\mathrm{dy}/dt=y\left(E_{11}-{\bar{E}}_{1}\right)=y\left(y-1\right)\\ \;\left[x\left(1-z\right)\left(-L-C_{4}\right)-xA+C_{2}-xP\right] \end{array}$$

If $z=\frac{xA-C_{2}+xP+xL+xC_{4}}{xL+xC_{4}}$, $\frac{dF\left(y\right)}{\mathrm{dy}}\text{≡}0$, it is stable regardless of the value of y. If $z\neq \frac{xA-C_{2}+xP+xL+xC_{4}}{xL+xC_{4}}$, then it is in a steady state at y = 0 and y = 1.

Perform the derivation on $F\left(y\right)$:


$$\frac{dF\left(y\right)}{\mathrm{dy}}=\left(2x-1\right)\left[x\left(1-z\right)\left(-L-C_{4}\right)-xA+C_{2}-xP\right]$$

When $z>\frac{xA-C_{2}+xP+xL+xC_{4}}{xL+xC_{4}}$,$\frac{dF\left(y\right)}{dt}|{\;}_{y=1}>0$, $\frac{dF\left(y\right)}{dt}|{\;}_{y=0}<0$, $y^{*}=0$ is an evolutionarily stable strategy. When $z<\frac{xA-C_{2}+xP+xL+xC_{4}}{xL+xC_{4}}$, $\frac{dF\left(y\right)}{dt}|{\;}_{y=1}<0$, $\frac{dF\left(y\right)}{dt}|{\;}_{y=0}>0$, $y^{*}=1$ is an evolutionarily stable strategy.

### 3.3 Evolutionary stabilization strategies for consumers

Consumer satisfaction and dissatisfaction expected returns $E_{31}$ and $E_{32}$, and average expected return ${\bar{E}}_{3}$ are:


$$E_{31}=xyR_{2}+y\left(1-x\right)R_{2}+\left(1-y\right)xR_{2}+\left(1-y\right)\left(1-x\right)R_{2}=R_{2}$$

$$\begin{array}{l} E_{32}=xy\left(-C_{4}\right)+y\left(1-x\right)\left(-T_{1}-C_{4}\right)+x\left(1-y\right)\\ \;\left(-C_{3}-C_{4}\right)+\left(1-x\right)\left(1-y\right)\left(-L-L_{1}-C_{3}\right) \end{array}$$

$${\bar{E}}_{3}=zE_{31}+\left(1-z\right)E_{32}$$

The replication dynamic equation for consumer strategy choice is:


$$\begin{array}{l} \begin{array}{l} F\left(z\right)=dz/dt=z\left(E_{31}-{\bar{E}}_{3}\right)=z\left(z-1\right)\\ \;(-C_{3}-L-L_{1}-R_{2}-C_{4}x+C_{3}y\\ \;-C_{4}y+Lx+L_{1}x+Ly+L_{1}y \end{array}\\ \;-T_{1}y+C_{4}xy-Lxy-L_{1}xy+T_{1}xy) \end{array}$$

If $y=\frac{C_{3}+L_{1}+L-R_{1}+xC_{4}-xL-xL_{1}}{C_{3}-C_{4}+L+L_{1}-T_{1}+xC_{4}-xL-xL_{1}+xT_{1}}$,$\frac{dF\left(z\right)}{dz}\text{≡}0$, it is stable regardless of the value of z. If $y\neq \frac{C_{3}+L_{1}+L-R_{1}+xC_{4}-xL-xL_{1}}{C_{3}-C_{4}+L+L_{1}-T_{1}+xC_{4}-xL-xL_{1}+xT_{1}}$, then it is in a steady state at z = 0 and z = 1.

Perform the derivation on $F\left(z\right)$:


$$\frac{dF\left(z\right)}{dz}=\left(2z-1\right)\left(\begin{array}{l} -C_{3}-L-L_{1}-R_{2}-C_{4}x+C_{3}y-C_{4}y\\ +Lx+L_{1}x+Ly+L_{1}y-T_{1}y+C_{4}xy\\ -Lxy-L_{1}xy+T_{1}xy \end{array}\right)$$

When $y>\frac{C_{3}+L_{1}+L-R_{1}+xC_{4}-xL-xL_{1}}{C_{3}-C_{4}+L+L_{1}-T_{1}+xC_{4}-xL-xL_{1}+xT_{1}}$, $\frac{dF\left(z\right)}{dt}|{\;}_{z=1}>0$, $\frac{dF\left(z\right)}{dt}|{\;}_{z=0}<0$, $z^{*}=0$is an evolutionarily stable strategy. When $y<\frac{C_{3}+L_{1}+L-R_{1}+xC_{4}-xL-xL_{1}}{C_{3}-C_{4}+L+L_{1}-T_{1}+xC_{4}-xL-xL_{1}+xT_{1}}$, $\frac{dF\left(z\right)}{dt}|{\;}_{z=1}<0$, $\frac{dF\left(z\right)}{dt}|{\;}_{z=0}>0$, $z^{*}=1$is an evolutionarily stable strategy.

### 3.4 Stability analysis

The system equilibrium point is $E_{1}\left(0,0,0\right)$, $E_{2}\left(0,0,1\right)$, $E_{3}\left(0,1,0\right)$, $E_{4}\left(1,0,0\right)$, $E_{5}\left(0,1,1\right)$, $E_{6}\left(1,0,1\right)$, $E_{7}\left(1,1,0\right)$, $E_{8}\left(1,1,1\right)$ from $F\left(x\right)=0$,$F\left(y\right)=0$,$F\left(z\right)=0$. The Jacobi matrix of the three-party evolutionary game $J=\left[\begin{array}{ccc} A_{11} & A_{12} & A_{13}\\ A_{21} & A_{22} & A_{23}\\ A_{31} & A_{32} & A_{33} \end{array}\right]$.


$$\begin{array}{l} A_{11}=x\left(C_{1}+L_{1}-R_{1}+Py+Ayz-1\right)+\\ \;\left(x-1\right)\left(C_{1}+L_{1}-R_{1}+Py+Ayz-1\right) \end{array}$$

$$A_{12}=x\left(P+Az\right)\left(x-1\right);A_{13}=Axy\left(x-1\right)$$

$$A_{21}=‐y\left(y-1\right)\left[A+P-\left(z-1\right)\left(C_{4}+L\right)\right]$$

$$\begin{array}{l} A_{22}=y\left[C_{2}-\mathrm{Ax}-Px+x\left(z-1\right)\left(C_{4}+L\right)\right]\\ \;+\left(y-1\right)\left[C_{2}-\mathrm{Ax}-Px+x\left(z-1\right)\left(C_{4}+L\right)\right] \end{array}$$

$$\begin{array}{l} A_{23}=xy\left(y-1\right)\left(C_{4}+L\right);\\ A_{31}=z\left(z-1\right)\left(\begin{array}{l} L-C_{4}+L_{1}+C_{4}y\\ -Ly-L_{1}y+T_{1}y \end{array}\right) \end{array}$$

$$A_{32}=z\left(z-1\right)\left(C_{3}-C_{4}+L+L_{1}-T_{1}+C_{4}x-Lx-L_{1}x+T_{1}x\right)$$

$$\begin{array}{l} A_{33}=-(z-{\mathrm{1)(C}}_{3}\;{\mathrm{+ L + L}}_{1}\;+\;R_{2}\;+\;C_{4}x-C_{3}y\;\\ +\;C_{4}y\;-\;Lx-L_{1}x-Ly-L_{1}{\mathrm{y + T}}_{1}y-C_{4}xy\theta \\ \;{\mathrm{+ Lxy + L}}_{1}xy-T_{1}xy)-z(C_{3}\;{\mathrm{+ L + L}}_{1}\;+\;R_{2}\;\\ +\;C_{4}y-C_{3}{\mathrm{y + C}}_{4}y-Lx-L_{1}x-Ly-L_{1}y\;\\ +T_{1}y-C_{4}{\mathrm{xy + Lxy + L}}_{1}xy-T_{1}xy) \end{array}$$

Substituting the eight equilibrium points into the Jacobi matrix, respectively, the corresponding eigenvalues of each equilibrium point can be obtained, as shown in Table 3. According to the evolutionary game theory, the equilibrium point that satisfies the Jacobi matrix when all the eigenvalues are negative is the evolutionary equilibrium point.

**Table 3:** Eigenvalues of the Jacobi matrix.

Balance point	Eigenvalue 1	Eigenvalue 2	Eigenvalue 3
$E_{1}\left(0,0,0\right)$	$-C_{1}-L_{1}+R_{1}+1$	$-C_{2}$	$C_{3}+L+L_{1}+R_{2}$
$E_{2}\left(0,0,1\right)$	$-C_{1}-L_{1}+R_{1}+1$	$-C_{2}$	$-C_{3}-L-L_{1}-R_{2}$
$E_{3}\left(0,1,0\right)$	$-C_{1}-L_{1}+R_{1}-P+1$	$C_{2}$	$R_{2}+C_{4}+T_{1}$
$E_{4}\left(1,0,0\right)$	$C_{1}+L_{1}-R_{1}-1$	$-C_{2}+A+P+C_{4}+L$	$C_{3}+R_{2}+C_{4}$
$E_{5}\left(0,1,1\right)$	$-C_{1}-L_{1}+R_{1}-P+A-1$	$C_{2}$	$-R_{2}-2C_{4}-T_{1}$
$E_{6}\left(1,0,1\right)$	$C_{1}+L_{1}-R_{1}-1$	$-C_{2}+A+P$	$-C_{3}-R_{2}$
$E_{7}\left(1,1,0\right)$	$C_{1}+L_{1}-R_{1}+P-1$	$C_{2}-A-P-C_{4}-L$	$R_{2}+C_{4}$
$E_{8}\left(1,1,1\right)$	$C_{1}+L_{1}-R_{1}+P+A-1$	$C_{2}-A-P$	$-R_{2}-C_{4}$

Scenario 1 When$-C_{1}-L_{1}+R_{1}+1<0$, the trust loss of the government choosing not to regulate is smaller than the cost of choosing to regulate. The punishment of the government that the agricultural product producer chooses not to build the digital agricultural product traceability system is smaller than the cost of choosing to build it. The benefit the consumer gets when satisfied is more significant than the cost of defending his rights when dissatisfied. At this point, as can be seen from Table 4, the eigenvalues of the Jacobian matrix corresponding to the equilibrium point $E_{2}\left(0,0,1\right)$ are all less than 0. The equilibrium point $E_{2}\left(0,0,1\right)$ is stable, and its corresponding evolutionary strategy is (Not regulate, Not build, Satisfied).

**Table 4:** Stability analysis of the equilibrium solution.

Balance point	Eigenvalue symbol	Stability
$E_{1}\left(0,0,0\right)$	Having positive value	Unsteady point
$E_{2}\left(0,0,1\right)$	When $-C_{1}-L_{1}+R_{1}+1<0$, all negative values	ESS
$E_{3}\left(0,1,0\right)$	Having positive value	Unsteady point
$E_{4}\left(1,0,0\right)$	Having positive value	Unsteady point
$E_{5}\left(0,1,1\right)$	Having positive value	Unsteady point
$E_{6}\left(1,0,1\right)$	When $C_{1}+L_{1}-R_{1}-1<0$ and $-C_{2}+A+P<0$, all negative values	ESS
$E_{7}\left(1,1,0\right)$	Having positive value	Unsteady point
$E_{8}\left(1,1,1\right)$	When $C_{1}+L_{1}-R_{1}+P+A-1<0$ and $C_{2}-A-P<0$, all negative values	ESS

Scenario 2 When $C_{1}+L_{1}-R_{1}-1<0$and$-C_{2}+A+P<0$, the cost to the government of choosing to regulate is less than the benefit of choosing to regulate. The penalties imposed on agricultural producers for choosing not to build a traceability system are greater than the sum of the benefits and incentives of choosing to build one. When satisfied, the benefits of food safety to consumers are more significant than the costs of defending their rights when dissatisfied. At this point, as can be seen from Table 4, the eigenvalues of the Jacobian matrix corresponding to the equilibrium point $E_{6}\left(1,0,1\right)$ are all less than 0. The equilibrium point $E_{6}\left(1,0,1\right)$ is stable, and its corresponding evolutionary strategy is (Regulate, Not build, Satisfied).

Scenario 3 When $C_{1}+L_{1}-R_{1}+P+A-1<0$ and $C_{1}+L_{1}-R_{1}+P+A-1<0$, the cost to the government of choosing to regulate is less than the benefit it receives from choosing to regulate, and the cost to agricultural producers of constructing a digital traceability system is less than the penalties imposed by the government in the event of consumer dissatisfaction. At this point, as can be seen from Table 4, the eigenvalues of the Jacobian matrix corresponding to the equilibrium point $E_{8}\left(1,1,1\right)$ are all less than 0. The equilibrium point $E_{8}\left(1,1,1\right)$ is stable, and its corresponding evolutionary strategy is (Regulate, Build, Satisfied).

## 4 Simulation analysis

**Figure 2:**
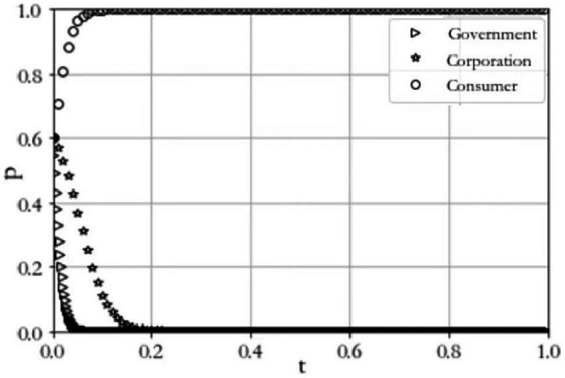
Evolutionary outcome of scenario 1.

Scenario 1 $-C_{1}-L_{1}+R_{1}+1<0$. When $C_{1}=8$, $R_{1}=6$, $L_{1}=4$, $C_{2}=6$, A=5, P=3, $R_{2}=7$, $C_{3}=2$, $C_{4}=6$, L=4. The results of the simulation experiment are shown in Figure 2. As can be seen from the figure, the probability that the government chooses to regulate keeps decreasing until it is 0; the probability that the agricultural producer chooses to construct keeps decreasing until it is 0; and the probability that the consumer is satisfied keeps increasing until it is 1. Therefore, the evolutionary equilibrium state tends to be (0,0,1). In this case, consumers choose not to file complaints due to the high cost of defending their rights, and the probability of government regulation will be very low, which makes agricultural producers not to build a digital traceability system.

**Figure 3:**
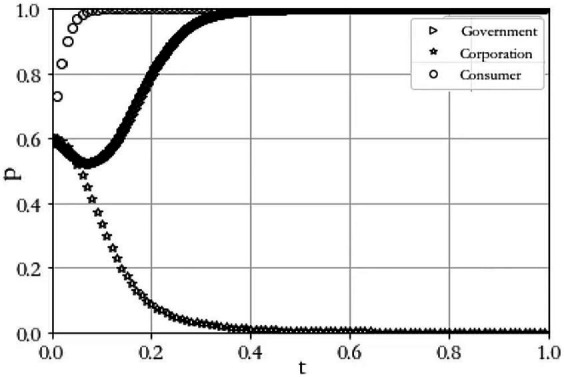
Evolutionary outcome of scenario 2.

Scenario 2 $C_{1}+L_{1}-R_{1}-1<0$ and $-C_{2}+A+P<0$. When $C_{1}=8$, $R_{1}=9$, $L_{1}=4$, $C_{2}=8$, A=5, P=3, $R_{2}=7$, $C_{3}=2$, $C_{4}=6$, L=4. The results of the simulation experiment are shown in Figure 3. From the figure, it can be seen that the probability that the government chooses to regulate keeps rising until it is 1; the probability that the agricultural producer chooses to construct keeps falling until it is 0; and the probability that the consumer is satisfied keeps rising until it is 1, so the evolutionary equilibrium state tends to (1, 0, 1). Compared with Case 1, the benefit $R_{1}$ gained from the regulation of agricultural product producers increases, and the probability of their choosing to regulate increases, the cost $C_{2}$ of agricultural product producers constructing a digital traceability system increases, and even though the government will penalize them, the cost of constructing a digital traceability system is higher than that of penalizing them, so that agricultural product producers will still tend to choose not to construct it. The consumer cannot obtain the quality traceability information of agricultural products they purchase, and the cost of consumers defending their rights is high, so the probability that the consumer chooses to file a complaint decreases.

**Figure 4:**
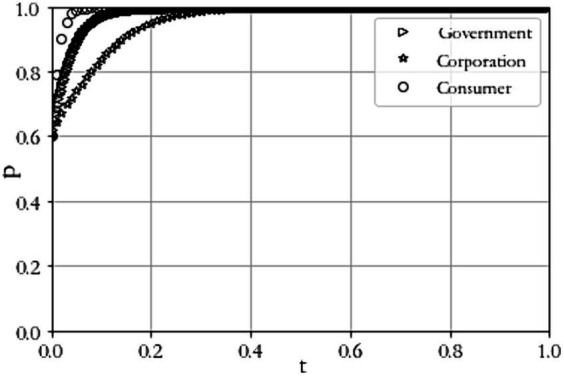
Evolutionary outcome of scenario 3.

Scenario 3 $C_{1}+L_{1}-R_{1}+P+A-1<0$ and $C_{2}-A-P<0$. When $C_{1}=8$, $R_{1}=9$, $L_{1}=4$, $C_{2}=6$, A=5, P=7, $R_{2}=7$, $C_{3}=2$, $C_{4}=6$, L=4. The results of the simulation experiment are shown in Figure 4. From the figure, it can be seen that the probability of the government choosing to regulate has been rising until it is 1; the probability of the agricultural production enterprise choosing to build has been rising until it is 1; the probability of the consumer being satisfied has been rising until it is 1, so the evolutionary equilibrium state tends to (1). Compared to Case 1, the gain $R_{1}$ gained from government regulation increases, the probability of its regulation increases, the penalty P for not constructing a digital traceability system for agricultural product producers increases, the probability of agricultural product producers constructing it increases, consumers can obtain traceability information about their products, agricultural product producers tend to produce high-quality products, and the probability of consumer satisfaction increases.

## 5 Conclusion and recommendation

This paper constructs a three-party evolutionary game model among the government, agricultural product producers, and consumers in the digital environment, analyzes the evolutionary process of agricultural product quality and safety issues involving the government, agricultural product producers, and consumers, and studies the strategic choices of the government, agricultural product producers and consumers as well as their influencing factors through the analysis of arithmetic examples. It is found that there are multiple evolutionary stabilization strategies in the tripartite evolutionary game model of agricultural product quality and safety regulation. When the benefits gained from government regulation, the cost of constructing a digital traceability system for agricultural product producers, and the penalties imposed on agricultural product producers are constantly changing, there will be (Not regulate, Not build, Satisfied), (Regulate, Not build, Satisfied), and (Regulate, Build, Satisfied) in order.

From this, the following recommendations can be drawn: (1) The government should set appropriate penalties and impose severe penalties for agricultural producers who violate production standards and quality requirements to force agricultural producers to improve quality (53). (2) Reduce the cost of enterprises to build a digital traceability system for agricultural products (54). The government can provide relevant financial support and reduce or waive relevant taxes and fees, etc., to reduce the cost of building a digital agricultural product traceability system for agricultural product production enterprises and encourage more enterprises to join the ranks of digital management (3). Actively guide consumers’ understanding of the quality and safety traceability of agricultural products and teach them to inquire about product origin and quality information through the traceability system (55, 56). Advocating that priority be given to agricultural products with digital quality and safety traceability marks to increase consumers’ confidence and sense of security in purchasing. Consumers should also actively report suspected agricultural product quality problems to government regulators and actively participate in supervision and public opinion monitoring to safeguard the quality and safety of agricultural products. This study also has some limitations. In the next step, we will expand the scope of the study to include third-party testing organizations and the public, and explore the characteristics and laws of multi-body behavioral decision-making in agricultural product quality and safety supervision. In addition, the game model we chose has a bias in describing the real decision-making environment, and we will also choose a more appropriate model to improve our study in the subsequent research.

## Data availability statement

The original contributions presented in the study are included in the article/supplementary material, further inquiries can be directed to the corresponding author.

## Author contributions

HH: Conceptualization, Formal analysis, Resources, Validation, Writing – original draft, Writing – review & editing. XL: Conceptualization, Data curation, Funding acquisition, Investigation, Methodology, Resources, Software, Supervision, Validation, Visualization, Writing – original draft, Writing – review & editing.
